# Editorial Board Members’ Collection Series: Gastrointestinal and Hepatic Diseases

**DOI:** 10.3390/medicina61040758

**Published:** 2025-04-20

**Authors:** Ludovico Abenavoli, Marcello Candelli

**Affiliations:** 1 Department of Health Sciences, University “Magna Graecia”, Viale Europa, 88100 Catanzaro, Italy; 2 Emergency, Anesthesiological and Reanimation Sciences Department of Fondazione Policlinico Agostino Gemelli—IRCCS, Catholic University of Rome, Largo Agostino Gemelli 8, 00168 Rome, Italy; marcello.candelli@policlinicogemelli.it

The Special Issue titled “Editorial Board Members’ Collection Series: Gastrointestinal and Hepatic Diseases” is a collection of papers from our Editorial Board Members and researchers invited by them. The aim is to provide a venue for networking and communication between *Medicina* and scholars in the field of gastrointestinal and hepatic diseases. Recent studies have highlighted significant advancements in understanding various medical conditions through innovative biomarkers, novel therapeutic strategies, and epidemiological insights. One of the key findings involves hemogram-derived ratios, such as platelet-to-lymphocyte and platelet-to-neutrophil ratios, which have emerged as potential tools for differentiating between idiopathic and secondary pulmonary fibrosis. This approach, along with hepatic biomarkers, could refine diagnostic accuracy and improve disease management [1]. The interplay between thyroid function and liver cirrhosis has also gained attention, with research suggesting that thyroid-stimulating hormone levels may correlate with disease severity and prognosis. Similarly, the impact of Coronavirus Disease-19 on acute cholangitis has been explored, revealing extended hospital stays, a higher prevalence of malignant causes, and notable shifts in microbial infections. In oncology, the increasing incidence of malignant polyps in younger populations has prompted a reconsideration of colorectal cancer screening guidelines [2]. The growing recognition of shifting tumor biology underscores the need for earlier interventions. Meanwhile, treatment strategies for inflammatory bowel diseases (IBD) continue to evolve, with subcutaneous vedolizumab proving effective in maintaining remission after intravenous therapy [3]. The relationship between metabolic disorders and gastrointestinal diseases remains a critical area of research. In this regard, non-alcoholic fatty liver disease appears increasingly prevalent among individuals with IBD, particularly those with ulcerative colitis. Given the close link between metabolic dysfunction and liver disease, experts emphasize the importance of comprehensive management strategies targeting cardiometabolic risk factors [4]. Beyond metabolic disorders, new perspectives are emerging on the potential connections between *Helicobacter pylori* infection and coronary artery disease, highlighting the role of chronic inflammation in cardiovascular health. Dietary habits, particularly those influenced by the Western diet and food additives, are also being scrutinized for their contribution to metabolic dysfunction-associated steatotic liver disease [5]. Advances in gastrointestinal research extend to conditions such as irritable bowel syndrome and slow transit constipation, both of which significantly affect quality of life [6]. The gut–brain axis, microbiome imbalances, and potential therapeutic interventions, including probiotics and fecal microbiota transplantation, are shaping new treatment paradigms. Similarly, the management of hepatic hemangiomas has shifted toward minimally invasive procedures, reflecting broader trends in interventional medicine [7]. Ongoing research into biofilm formation in biliary stents and the challenges of cirrhotic cardiomyopathy further illustrate the complexity of modern medical science. As diagnostic and therapeutic approaches continue to advance, integrating these findings into clinical practice will be crucial for improving patient outcomes across a wide range of diseases [8]. Gastroenterology is a rapidly advancing field that comprises both pre-clinical and clinical areas. The development of new treatments, cutting-edge technologies, and a deeper understanding of disease mechanisms are key factors driving this progress. In this regard, this Research Topic provides scholars with a comprehensive and current perspective, emphasizing the latest innovations and developments in the pursuit of global excellence in gastroenterology.

## References

[1] Paluschinski M., Loosen S., Kordes C., Keitel V., Kuebart A., Brandenburger T., Schöler D., Wammers M., Neumann U.P., Luedde T. (2023). Extracellular Vesicles as Markers of Liver Function: Optimized Workflow for Biomarker Identification in Liver Disease. Int. J. Mol. Sci..

[2] Tanadi C., Tandarto K., Stella M.M., Sutanto K.W., Steffanus M., Tenggara R., Bestari M.B. (2023). Colorectal cancer screening guidelines for average-risk and high-risk individuals: A systematic review. Rom. J. Intern. Med..

[3] Crooks B., Barnes T., Limdi J.K. (2020). Vedolizumab in the treatment of inflammatory bowel disease: Evolving paradigms. Drugs Context.

[4] Abenavoli L., Spagnuolo R., Scarlata G.G.M., Scarpellini E., Boccuto L., Luzza F. (2023). Ultrasound Prevalence and Clinical Features of Nonalcoholic Fatty Liver Disease in Patients with Inflammatory Bowel Diseases: A Real-Life Cross-Sectional Study. Medicina.

[5] Armandi A., Bugianesi E. (2024). Dietary and pharmacological treatment in patients with metabolic-dysfunction associated steatotic liver disease. Eur. J. Intern. Med..

[6] Cassar G.E., Youssef G.J., Knowles S., Moulding R., Austin D.W. (2020). Health-Related Quality of Life in Irritable Bowel Syndrome: A Systematic Review and Meta-analysis. Gastroenterol. Nurs..

[7] Loh J.S., Mak W.Q., Tan L.K.S., Ng C.X., Chan H.H., Yeow S.H., Foo J.B., Ong Y.S., How C.W., Khaw K.Y. (2024). Microbiota-gut-brain axis and its therapeutic applications in neurodegenerative diseases. Signal Transduct. Target. Ther..

[8] Prasad S.S., Potter M., Keely S., Talley N.J., Walker M.M., Kairuz T. (2019). Roles of healthcare professionals in the management of chronic gastrointestinal diseases with a focus on primary care: A systematic review. JGH Open.

